# Symptomatic Characteristics of Hypozincemia Detected in Long COVID Patients

**DOI:** 10.3390/jcm12052062

**Published:** 2023-03-06

**Authors:** Yui Matsuda, Kazuki Tokumasu, Yuki Otsuka, Naruhiko Sunada, Hiroyuki Honda, Yasue Sakurada, Yasuhiro Nakano, Toru Hasegawa, Mikako Obika, Keigo Ueda, Fumio Otsuka

**Affiliations:** Department of General Medicine, Okayama University Graduate School of Medicine, Dentistry and Pharmaceutical Sciences, Okayama 700-8558, Japan

**Keywords:** dysgeusia, dysosmia, fatigue, hypozincemia, long COVID

## Abstract

Objectives: The aim of this study was to determine the characteristics of hypozincemia in long COVID patients. Methods: This study was a single-center retrospective observational study for outpatients who visited the long COVID clinic established in a university hospital during the period from 15 February 2021 to 28 February 2022. Characteristics of patients with a serum zinc concentration lower than 70 μg/dL (10.7 μmol/L) were compared with characteristics of patients with normozincemia. Results: In a total of 194 patients with long COVID after excluding 32 patients, hypozincemia was detected in 43 patients (22.2%) including 16 male patients (37.2%) and 27 female patients (62.8%). Among various parameters including the background characteristics of the patients and medical histories, the patients with hypozincemia were significantly older than the patients with normozincemia (median age: 50 vs. 39 years). A significant negative correlation was found between serum zinc concentrations and age in male patients (*R* = −0.39; *p* < 0.01) but not in female patients. In addition, there was no significant correlation between serum zinc levels and inflammatory markers. General fatigue was the most frequent symptom in both male patients with hypozincemia (9 out of 16: 56.3%) and female patients with hypozincemia (8 out of 27: 29.6%). Patients with severe hypozincemia (serum zinc level lower than 60 μg/dL) had major complaints of dysosmia and dysgeusia, which were more frequent complaints than general fatigue. Conclusions: The most frequent symptom in long COVID patients with hypozincemia was general fatigue. Serum zinc levels should be measured in long COVID patients with general fatigue, particularly in male patients.

## 1. Introduction

The coronavirus disease 2019 (COVID-19) pandemic, caused by severe acute respiratory syndrome coronavirus 2 (SARS-CoV-2) [1], has led to over 634 million cases of infection and over 6.6 million deaths worldwide since April 2020. In addition to the acute symptoms of COVID-19, COVID-19 also causes prolonged sequelae, which are referred to as post COVID-19 condition (PCC), post-acute sequelae of severe acute respiratory syndrome coronavirus 2 (SARS-CoV-2), or long COVID [2].

Long COVID has been reported to be present in at least one-third of COVID-19 patients at 60 days after onset of COVID-19, and its symptoms include fatigue, dysosmia, dysgeusia, headache, hair loss, dyspnea, dizziness, memory loss, cognitive impairment and insomnia [3,4,5,6]. These systemic and multisystemic symptoms of long COVID are similar to hypozincemia in some aspects [7,8], and zinc is also attracting attention as a treatment for neuropsychiatric symptoms in COVID-19 patients [9]. Hypozincemia has been suggested to be a possible etiology of smell and taste alterations in COVID-19 patients [10].

Although a relatively large number of patients suffer from various prolonged symptoms due to COVID-19, the pathophysiology and prognosis of long COVID have not been clarified, and specific treatments for long COVID have not been established. There have been few studies focusing on the relationship between hypozincemia with the manifestation of various symptoms of long COVID. In this study, we investigated the clinical characteristics of hypozincemic conditions in long COVID patients.

## 2. Patients and Methods

### 2.1. Study Design and Patients’ Characteristics

This study was a retrospective observational study conducted in a single facility. We reviewed the medical records of patients who visited our COVID-19 aftercare outpatient clinic (CAC) during the period from 15 February 2021 to 28 February 2022. Our CAC was established on 15 February 2021 in the Department of General Medicine, Okayama University Hospital. The purpose of the CAC is to evaluate and treat patients suffering from long COVID symptoms. Long COVID was defined as symptoms that persist for more than four weeks after the onset of COVID-19 [2,11,12]. All of the long COVID patients who were more than twelve years of age and who had visited the CAC during the study period were included in this study. We acquired information on age, sex, body mass index (BMI), current smoking and alcohol drinking habits, admission due to COVID-19, therapeutic use of oxygen in the acute phase, severity of COVID-19, history of COVID-19 vaccination, duration after onset of COVID-19 to the first visit and clinical symptoms of long COVID. The severity of the acute phase of COVID-19 was determined on the basis of the criteria defined by the Ministry of Health, Labour and Welfare in Japan [13]. Clinical symptoms of long COVID were identified by a face-to-face medical interview with a physician.

### 2.2. Determination of Serum Zinc Deficiency

To determine serum zinc concentrations, we used Accuras Auto Zn (Shino-Test Corporation, Tokyo, Japan), a test kit for pharmaceuticals for external diagnosis, using an automatic analyzer in the Clinical Laboratory in our hospital. The intra-assay and inter-assay coefficients of variation for zinc concentration were below 10% and 15%, respectively, and the limit of quantification was 2.0 μg/dL. In Harrison’s Principles of Internal Medicine, which is used as an international standard reference, clinical zinc deficiency is defined as a serum zinc level <70 μg/dL (10.7 μmol/L) [14]. Since serum zinc levels tend to be affected by age, sex, and the time of blood sampling, several cutoff values have been proposed for various situations [15,16]. However, in the present study, a zinc deficiency condition was set at a zinc level of <70 μg/dL. Since zinc deficiency is defined as a zinc level less than 60 μg/dL in some reports [17], zinc levels of less than 60 μg/dL were defined as severe zinc deficiency in this study. According to the Japanese Society of Clinical Nutrition, a serum zinc concentration up to 130 μg/dL is normal, and patients with serum zinc levels >130 μg/dL were therefore excluded from the analysis. Patients who were already taking zinc medications and those who did not have any blood examinations for the determination of the serum zinc level were also excluded from the analysis.

### 2.3. Statistical Analysis

All statistical analyses were conducted by using Stata/SE 17.0 (StataCorp, College Station, TX, USA). The characteristics of hypozincemia patients and those of normozincemic patients were compared using Pearson’s χ^2^ tests for categorical variables because there was an adequate sample size. The data were also analyzed using linear regression analysis and Spearman’s rank correlation coefficients to determine interrelationships between parameters. Mann–Whitney U tests were used for continuous variables with non-normal distributions, and Student’s *t*-tests were used for continuous variables with normal distributions. Skewness and kurtosis tests were used to determine the normality of each variable. *p* values of less than 0.05 were considered statistically significant.

### 2.4. Ethical Approval

Information about the present study was provided on our hospital wall and website, and patients who requested to opt out were offered that opportunity. Informed consent was not required from the patients due to the anonymization of data. This study complied with the Ethics Committee of Okayama University Hospital (No. 2105-030) and adhered to the Declaration of Helsinki.

## 3. Results

Data for the patients who visited our CAC during the study period were obtained from medical records. Of the two hundred twenty-six patients who were enrolled, twenty-three patients who were already taking zinc medications, eight patients who did not have a blood examination and one patient with a serum zinc concentration above 130 μg/dL were excluded. Data for 194 patients who visited our CAC were used for analysis in this study.

The background characteristics of patients with long COVID in the hypozincemia group (serum zinc concentration <70 μg/dL) and the normozincemia group (serum zinc concentration ≥70 μg/dL) are shown in Table 1. Hypozincemia was detected in 43 (22.2%) of the 194 patients with long COVID. The hypozincemia group included 16 male patients (37.2%) and 27 female patients (62.8%). The median ages of the patients were 50 years (interquartile range [IQR]: 38.5–57.5 years) in the hypozincemia group and 39 years (IQR: 26.5–50 years) in the normozincemia group. Patients in the hypozincemia group were significantly older than those in the normozincemia group (*p* = 0.0051). Other parameters including BMI and the percentage of patients with a smoking habit and an alcohol drinking habit were not significantly different between the two groups. In addition, the percentage of patients with a hospital admission during the acute phase and the percentage of patients who had received oxygen supplementation were not significantly different between the two groups. The patients with hypozincemia included 27 patients (62.8%) who did not receive any vaccination.

**Figure 1:**
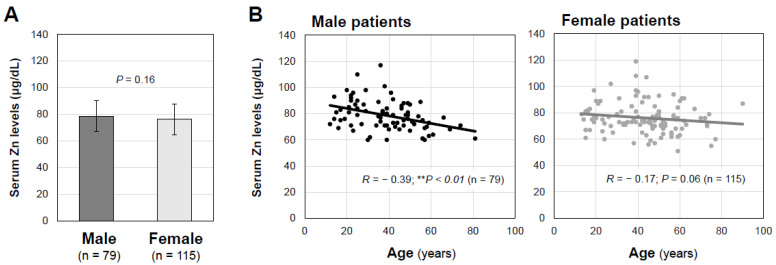
Serum zinc concentrations and age-dependent changes in serum zinc concentrations in male and female patients with long COVID. (**A**) Serum zinc concentrations (μg/dL) are shown as means ± SD in male patients (*n* = 79) and female patients (*n* = 115) with long COVID. (**B**) Interrelationships between serum zinc concentrations and patients’ ages are shown as scatter diagrams for male patients (*n* = 79) and female patients (*n* = 115). ** *p* < 0.01 indicates a statistically significant difference.

**Figure 2:**
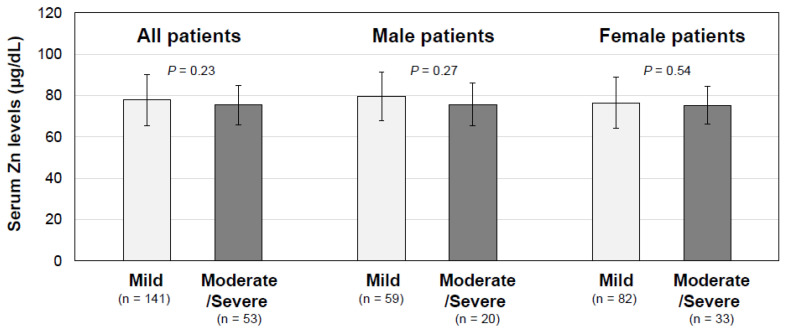
Serum zinc concentrations based on the severity of the acute phase of COVID-19. Serum zinc levels (μg/dL) are shown as means ± SD in three categories based on the severity of the acute phase of COVID-19. The overall group includes 141 mild cases and 53 moderate-to-severe cases, the male patients group includes 59 mild cases and 20 moderate-to-severe cases and the female patients group includes 82 mild cases and 33 moderate-to-severe cases.

**Figure 3:**
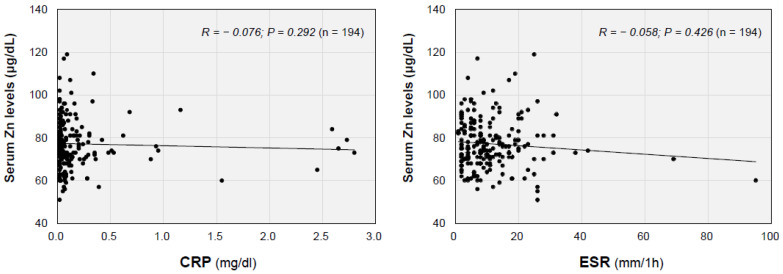
Serum zinc concentrations and inflammatory markers in the patients with long COVID. Interrelationships between the serum zinc concentration and serum C-reactive protein (CRP) level and the erythrocyte sedimentation rate (ESR) are shown as scatter diagrams (*n* = 194).

As shown in Figure 1A, the mean serum zinc concentrations were not significantly different in the male and female long COVID patients (*p* = 0.1556). As for the age-related changes in the serum zinc concentrations, the serum zinc concentrations in male patients significantly decreased in an age-dependent manner (*R* = −0.3937; *p* = 0.0003; *n* = 79), whereas no significant correlation between age and serum zinc concentration was found in the female patients (*R* = −0.1742; *p* = 0.0625; *n* = 115), as shown in Figure 1B. In the hypozincemia group, thirty patients (69.8%), eleven patients (25.6%) and two patients (4.6%) had mild, moderate and severe disease in the acute phase of COVID-19, respectively (Table 1). However, serum zinc levels in the long COVID patients were not significantly different among the disease severity groups in all of the patients (*p* = 0.230) and in male patients (*p* = 0.2738) and female patients (*p* = 0.5443) (Figure 2). The serum zinc level was not significantly correlated with the serum C-reactive protein (CRP) level (*R* = −0.0761; *p* = 0.292; *n* = 194) or erythrocyte sedimentation rate (*R* = −0.0575; *p* = 0.426; *n* = 194) (Figure 3).

**Figure 4:**
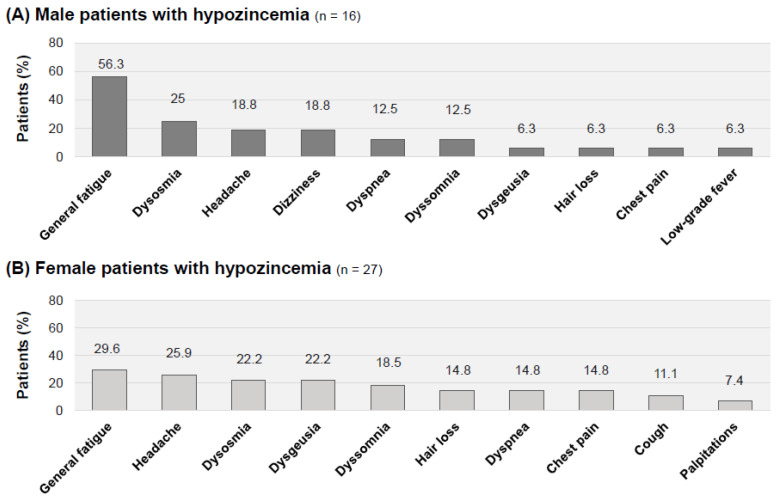
Gender-related symptoms of long COVID in patients with hypozincemia (<70 μg/dL). (**A**) Proportion of patients complaining of each symptom in long COVID male patients with a serum zinc concentration less than 70 μg/dL (*n* = 16). (**B**) Proportion of patients complaining of each symptom in long COVID female patients with a serum zinc concentration less than 70 μg/dL (*n* = 27). Symptoms are shown in order of frequency.

Gender-dependent symptoms in long COVID patients with hypozincemia are shown in Figure 4. General fatigue was the most frequent symptom in both the male patients with hypozincemia (nine patients: 56.3%) and female patients with hypozincemia (eight patients: 29.6%). The relatively frequent symptoms in the male patients with hypozincemia were dysosmia (four patients: 25%), headache (three patients: 18.8%), dizziness (three patients: 18.8%), dyspnea (two patients: 12.5%) and dyssomnia (two patients: 12.5%) (Figure 4A). On the other hand, the frequent symptoms in female patients with hypozincemia were headache (seven patients: 25.9%), dysosmia (six patients: 22.2%), dysgeusia (six patients: 22.2%) and dyssomnia (five patients: 18.5%) (Figure 4B).

**Figure 5:**
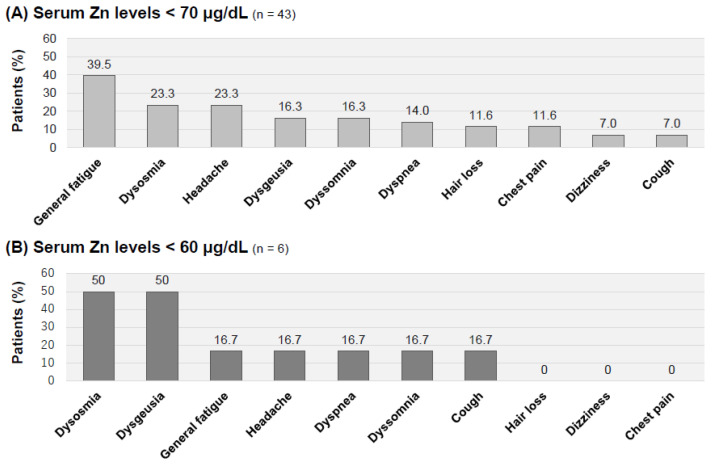
Symptomatic characteristics of long COVID patients with hypozincemia. (**A**) Proportion of patients complaining of each symptom in long COVID patients with a serum zinc concentration less than 70 μg/dL (*n* = 43). (**B**) Proportion of patients complaining of each symptom in long COVID patients with a serum zinc concentration less than 60 μg/dL (*n* = 6). Symptoms are shown in order of frequency.

The major complaints in patients with moderate hypozincemia and patients with severe hypozincemia are shown in Figure 5. The most frequent symptoms in the patients with moderate hypozincemia (serum zinc concentrations <70 μg/dL; *n* = 43) were general fatigue (seventeen patients: 39.5%), dysosmia (ten patients: 23.3%), headache (ten patients: 23.3%), dysgeusia (seven patients: 16.3%) and dyssomnia (seven patients: 16.3%), as shown in Figure 5A.

Moreover, by specifically focusing on the symptoms in long COVID patients with severe hypozincemia (serum zinc concentrations < 60 μg/dL; *n* = 6), it was revealed that the most frequent symptoms were dysosmia (three patients: 50%) and dysgeusia (three patients: 50%), which were more frequent than the other symptoms of general fatigue (one patient: 16.7%), headache (one patient: 16.7%), dyspnea (one patient: 16.7%), dyssomnia (one patient: 16.7%) and cough (one patient: 16.7%) (Figure 5B).

## 4. Discussion

The present study revealed the characteristics of clinical symptoms in long COVID patients with hypozincemia and showed the clinical utility of serum zinc examination in long COVID patients. General fatigue was the most frequent long COVID symptom followed by dysosmia and headache in patients with moderate hypozincemia defined as a serum zinc concentration below 70 μg/dL, which is a cut-off value for the serum zinc concentration as hypozincemia used in several studies [14,18]. On the other hand, the most frequent symptoms were dysosmia and dysgeusia in patients with severe hypozicemia, defined as a serum zinc concentration below 60 μg/dL, which is defined as zinc deficiency in the Japanese guidelines [17]. Furthermore, serum zinc concentrations decreased with aging, especially in male patients.

It has been reported that severe disease in the acute phase of COVID-19 was associated with lower zinc concentrations [18]. For instance, a decreased serum zinc concentration at admission was shown to be correlated with a higher mortality rate in the acute phase of COVID-19 [19] and also to be associated with a prolonged admission period [20]. Zinc is an essential element for the host as well as pathogens, and plasma zinc levels are generally reduced via the induction of inflammatory cytokines that upregulate the hepatic accumulation of zinc, leading to hypozincemia [21]. Serum zinc concentrations are known to be affected by inflammatory status [22], and a negative correlation between the serum levels of zinc and CRP has been reported in the patients with acute COVID-19 [23]; however, in the present study, there was no significant interrelationship between the degree of persistent inflammation and zinc depletion in the long COVID patients.

It seems that a deficiency in systemic zinc can lead to significant problems in nutrition and clinical medicine. Zinc deficiency has also been shown to be related to growth retardation, male hypogonadism, skin changes, hair loss, poor appetite, mental lethargy, delayed would healing, taste abnormalities, smell dysfunction, abnormal dark adaptation and anergic conditions [7,8,24]. Therefore, it is reasonable that long COVID patients with hypozincemia had more complaints, including dysgeusia and hair loss, in addition to general fatigue, which is a common complaint in long COVID patients.

A recent report has shown that hypozincemia was more frequent in COVID-19 patients than in non-COVID-19 patients (27.6% vs. 11.4%) [25]. In the present study, hypozincemia was found in 43 (22.2%) of the 194 patients with long COVID, and the percentage was similar to that in the study that focused on early COVID-19 [25]. In a Japanese study in elderly outpatients, hypozincemia (a serum zinc concentration of less than 66 μg/dL) was detected in 19% of the patients [26]. Although there are no directly comparable data, the prevalence rate of hypozincemia (22.2%) in the present study for relatively young patients with long COVID (median age of 42 years) is likely to be higher than that in the general population in Japan. Additionally, prolonged hypozincemia has been shown to be a risk factor for severe COVID-19 [18]. The severity of initial infection was not directly associated with hypozincemia detected in long COVID patients in our study. However, our data showed that general fatigue was the most common symptom in long COVID patients with hypozincemia and that the most frequent symptoms in long COVID patients with a serum zinc concentration below 60 μg/dL were dysosmia and dysgeusia, which are typical symptoms seen in patients with hypozincemia, regardless of COVID-19 [17,27].

Among the various complaints caused by long COVID, the symptoms of headache, dyspnea and dyssomnia might be associated with a progressive type of long COVID, namely, myalgic encephalomyelitis/chronic fatigue syndrome (ME/CFS) that is related to post-COVID-19 conditions [28]. In this regard, we have shown that the general prevalence rate of ME/CFS was 16.8% in a retrospective study in 279 long COVID patients [28]. ME/CFS is a debilitating disorder that is characterized by general fatigue, post-exertional malaise, sleep disturbance, different types of pain and neurological/cognitive dysfunctions lasting for longer than six months [29,30,31,32] Since neurological and cognitive dysfunctions were suggested to be related to hypozincemia [7,9], it is possible that hypozincemia is one of the factors underlying post COVID-19-induced ME/CFS. In the present study, general fatigue was a major symptom in both male and female long COVID patients with hypozincemia, but male long COVID patients with hypozincemia had more frequent symptoms of headaches, dizziness and dyspnea. This might be one of the reasons why the proportion of male patients is relatively large in patients with post COVID-19-induced ME/CFS [28] compared with the proportion in ME/CFS studies in general [33].

The present study further demonstrated an inverse correlation between age and serum zinc levels in patients with long COVID, particularly in male patients. The gender-dependent changes in serum zinc levels might be due to the fact that approximately 60% of zinc is contained in muscle tissues [34], of which the mass decreases with aging. The decrease in the serum androgen level [35] and the decrease in cellular zinc concentration [36] with aging might also be involved in this mechanism. It is also known that zinc intake tends to decline with aging in Japan, with a peak between the ages of 30 to 59 years [17].

The current study contains several limitations. First, this study was a Japanese single-center retrospective study. Because all the subjects were referred patients, it is possible that patients with more severe and long-term symptoms were included in this study, rather than patients with primary or self-limiting symptoms. A multicenter prospective study is therefore needed to prove the causality of the relationship between hypozincemia and long COVID symptoms. Second, variations and comorbidities in the patients’ backgrounds were not considered, since all of the patients who visited our CAC were included. Third, the time of blood sampling was not minutely considered in this study. Further research and data accumulation are necessary to elucidate the zinc-related pathophysiology of long COVID and to determine the effectiveness of zinc supplementation for long COVID patients.

In conclusion, we revealed that hypozincemia was present in one fifth of long COVID patients and that general fatigue was the most common symptom with dysosmia, headache, dysgeusia and dyssomnia also being common symptoms in long COVID patients with hypozincemia. Examination of serum zinc levels in long COVID patients with a consideration of gender-related and age-related changes in serum zinc levels would provide important information for evaluating changes in nutrition and metabolism related to long COVID.

## Figures and Tables

**Table 1:** Clinical characteristics of 194 patients with long COVID.

	Total	Hypozincemia (Zn < 70 μg/dL)	Normozincemia (Zn ≥ 70 μg/dL)	*p* Value
Number of patients	194	43 (22.2%)	151 (77.8%)	
Serum zinc concentration (μg/dL), median (IQR)	75 (70–84)	63 (60.5–67)	79 (73–87)	<0.01 ^(b)^ **
Age, median (IQR)	42 (28–52)	50 (38.5–57.5)	39 (26.5–50)	<0.01 ^(a)^ **
Sex				
Male	79 (40.7%)	16 (37.2%)	63 (41.7%)	0.595 ^(c)^
Female	115 (59.3%)	27 (62.8%)	88 (58.3%)	
BMI, median (IQR)	23.5 (20.6–26.7)	23.7 (20.9–27.2)	23.1 (20.6–26.1)	0.588 ^(b)^
Smoking habit	79 (40.7%)	16 (37.2%)	63 (41.7%)	0.595 ^(c)^
Alcohol drinking habit	86 (44.3%)	22 (51.2%)	64 (42.4%)	0.307 ^(c)^
Admission	60 (30.9%)	16 (37.2%)	44 (29.1%)	0.312 ^(c)^
O_2_ therapy	28 (14.4%)	9 (20.9%)	19 (12.6%)	0.169 ^(c)^
Severity of COVID-19 in acute phase				
Mild	141 (72.7%)	30 (69.8%)	111 (73.5%)	0.173 ^(c)^
Moderate	50 (25.8%)	11 (25.6%)	39 (25.8%)	
Severe	3 (1.5%)	2 (4.6%)	1 (0.7%)	
COVID-19 vaccination				
None	117 (60.3%)	27 (62.8%)	90 (59.6%)	0.927 ^(c)^
1 dose	21 (10.8%)	4 (9.3%)	17 (11.3%)	
2 doses	55 (28.4%)	12 (27.9%)	43 (28.5%)	
3 doses	1 (0.5%)	0 (0%)	1 (0.6%)	
Duration after the onset of COVID-19 to the 1st visit, median (IQR)	86 (55.3–128)	80 (58.5–129.5)	88 (55.5–128)	0.528 ^(b)^

Medians [IQR: interquartile ranges] and percentages (%) are shown. BMI: body mass index, COVID-19: coronavirus disease 2019. The ^(a)^ Mann–Whitney U test, ^(b)^ Student’s *t*-test and ^(c)^ χ^2^ test were performed for the hypozincemia and normozincemia groups. ** *p* < 0.01 indicates statistically significant difference.

## Data Availability

Detailed data will be available if a request is sent to the corresponding author.

## Abbreviations

Body mass index (BMI); coronavirus disease 2019 (COVID-19); COVID-19 aftercare clinic (CAC); C-reactive protein (CRP); erythrocyte sedimentation rate (ESR); myalgic encephalomyelitis/chronic fatigue syndrome (ME/CFS); and post COVID-19 condition (PCC).
